# Investigation of FBMC-OQAM Equalization with Real Interference Prediction Algorithm Properties for MIMO Transmission Scheme

**DOI:** 10.3390/s23042111

**Published:** 2023-02-13

**Authors:** Vladimir O. Varlamov, Evgeniy M. Lobov, Elizaveta O. Lobova

**Affiliations:** Science and Research Department, Moscow Technical University of Communications and Informatics, 111024 Moscow, Russia

**Keywords:** FBMC-OQAM, HF, equalization, MIMO, self-interference

## Abstract

Increasing the data transfer rate is an urgent task in cellular, high-frequency (HF) and special communication systems. The most common way to increase the data rate is to expand the bandwidth of the transmitted signal, which is often achieved through the use of multitone systems. One such system is the filter bank multicarrier (FBMC). In addition, speed improvements are achieved using multi-input–multi-output (MIMO) systems. In this study, we developed an algorithm for equalizing FBMC signals with offset-QAM modulation (OQAM) with self-interference compensation due to its correlation properties in a MIMO channel with memory. An analytical derivation of the proposed algorithm and an analysis of the computational complexity are given. According to the results of simulation modeling and a comparative analysis of performance in terms of the bit error rate and error vector magnitude with solutions with similar computational complexity, a similar level of performance was shown compared to a more complex parallel multistage algorithm, and a better performance was demonstrated compared to a one-tap algorithm.

## 1. Introduction

The development of telecommunication systems is moving in the direction of increasing the efficiency of using time–frequency resources. To improve the spectral efficiency, new methods of multiplexing user data are being developed, such as non-orthogonal multiple access (NOMA), and multiple-input–multiple-output (MIMO) spatial multiplexing algorithms are being improved (Massive MIMO and MU-MIMO technologies). Higher-order manipulation is used, coupled with an increase in the signal-to-noise ratio (due to a decrease in the coverage area of base stations and access points), and new group signal structures are being developed that provide greater spectral efficiency compared to cyclic prefix orthogonal frequency division multiplexing (CP-OFDM) technology, which is the most common technology in radio data transmission systems requiring high transmission speeds. One of these technologies is the filter bank multicarrier (FBMC), which provides a lower level of out-of-band emissions by filtering signals on each subcarrier and enables an increase in spectral efficiency due to the possibility of eliminating the cyclic prefix, which makes this modulation technique a prospect for future cellular communication systems [1,2,3]. The idea of the FBMC system comes from attempts to change the time and frequency localization of generated discrete modulation pulses. Using perfect reconstruction filter bank theory, in [4], the authors introduced a data transmission system algorithm that allows the manipulation of the localization of data symbol pulses to achieve the higher spectral efficiency of the signal. The applicability of FBMC modulation for high-frequency (HF) channels was introduced in [5] with the addition of the spread-spectrum (SS) technique, and the robustness of the introduced modulation technology was proved for channels with low SNR values and a wide range of Doppler spread and delays. Further, the same authors in [6,7] provide theoretical and practical comparisons of the FBMC-SS system and Walsh sequence-based direct sequence spread systems (DSSS). The same authors in [8] presented an analysis of FBMC-SS performance in doubly dispersive HF channels and showed methods of system parameter optimization to reduce the influence of doubly dispersive channel distortions. In [9], another author group provides a comparison of three types of multicarrier modulation systems—CP-OFDM, offset-QAM FBMC (FBMC-OQAM) and filtered multitone (FMT). The authors concluded that the best performance was obtained with the filtered multitone system (based on the same filter bank paradigm, except using non-overlapping subcarriers and filters instead of FBMC) thanks to the null self-interference influence, but the authors note that benefits from increased FBMC spectral efficiency are more significant and emphasize the importance of channel estimation and equalization algorithms for FMBC-OQAM systems. Another comparison of FBMC-OQAM, CP-OFDM and FMT performance was presented in [9] and shows that CP-OFDM and FMT have advantageous effects only for high SNR values, where the FBMC-OQAM self-interference noise level became comparable to channel noise. In [10], we provide an investigation of FBMC-OQAM signal properties in conditions of wideband HF channels, and the presented modeling results show the resistance abilities of FBMC-OQAM signals in the case of Watterson’s channel model [11], including the influence of ionospheric dispersion distortions of signals, which greatly corrupt wideband signals.

As noted in the works discussed above, the development of algorithms for qualitative channel estimation under conditions of FBMC-OQAM self-interference is an important task for such systems. Solutions to this problem are based on the generation of a training sequence that will cancel the self-interference of each symbol and will be separated from the data payload to protect the training sequence from interference generated by payload symbols. In [12,13], the authors described a set of preambles for FBMC-OQAM MIMO systems based on interference approximation methods (IAMs) and an interference cancellation method (ICM). Another method [14], applicable for pilot symbols and preambles integrated into data payload, is to generate additional helping symbols, whose values will cancel self-interference from surrounding symbols. However, these methods can be applied to cancel self-interference only for a preamble or pilot signal, because they significantly decrease the spectral efficiency of transmitted data.

The above review shows that the FBMC-OQAM system shows good performance in distortion channels and is a candidate for high-speed, high-frequency ionospheric communication systems. However, this signal modulation technology introduces its own noise into the demodulated signal. When working with low-order modulations and low signal-to-noise ratios (SNRs), this interference does not have a significant effect on noise immunity, while for quadrature amplitude modulations (QAM) with higher orders, with SNRs at which the bit error probability begins to tend to zero, the level of intrinsic interference is such that it has a significant impact on the quality of signal reception.

The purpose of this work was to determine the statistical properties of the FBMC-OQAM modem’s self-interference in a MIMO channel with memory and to develop an equalization algorithm with self-interference compensation.

The basic idea of this type of equalization depends on the phenomenon of the presence of a correlation between the real and imaginary parts of the intrinsic interference of the FBMC-OQAM communication system in memory channels (equalization with real interference prediction—ERIP), which was discovered for the first time by the authors in [15], where the gain in noise immunity compared to a single-tap equalizer was shown by compensating for the real part of the interference with its prediction. Additionally, in this paper, the authors proved that this correlation depends on the modulator and demodulator structures, parameters and channel impulse response. Thus, these coefficients can be calculated once and used until the channel impulse response is updated. For this reason, this algorithm is interesting for communication systems working with relatively slowly changing channels, allowing the achievement of better signal quality performance with practically the same computational complexity as the one-tap algorithm. Later, the authors in [16] extended this method for doubly selective channels by taking into account the emerging correlation between adjacent subcarriers and showed their solution‘s performance by comparing it with equalizers with similar computational complexity. However, this approach eliminates the low computational complexity advantage, because it requires computing new correlation coefficients for each narrowband symbol. For this reason, we focus on the application of an interference prediction approach for time-independent channels.

## 2. FBMC-OQAM System Model

In this section, we present a brief mathematical description of the FBMC-OQAM system model used. The information transmission system implemented using filter bank technology is based on algorithms of multirate synthesis and analysis filter banks with perfect reconstruction, as presented in [17]. From a set of narrowband signals, just as in an OFDM demodulator, a wideband signal is divided into subcarrier signals using a Fourier transform. The synthesis algorithm, in turn, generates a wideband signal from a set of narrowband channel signals, while the perfect reconstruction (PR) conditions imposed on the prototype filter design imply that the conversion of a set of narrowband signals to a wideband one occurs without distortion. Ideal signal recovery will be achieved if the aliasing signal component after channel filtering is equal to zero (i.e., the part of the signal that remains unfiltered outside the passband):

(1) $$\begin{array}{cc} \sum_{\tau =-\infty }^{\infty }g\left[l+\tau \frac{M}{2}\right]g\left[l+\tau \frac{M}{2}+nM\right]=\delta \left(n\right) & \begin{array}{c} n=0,1,2,\ldots \\ l=0,1,2,\ldots ,M-1 \end{array} \end{array},$$

where g(k) is the real and symmetric impulse response (IR) of the prototype filter, and M is the number of channels of the filter bank.

**Figure 1:**
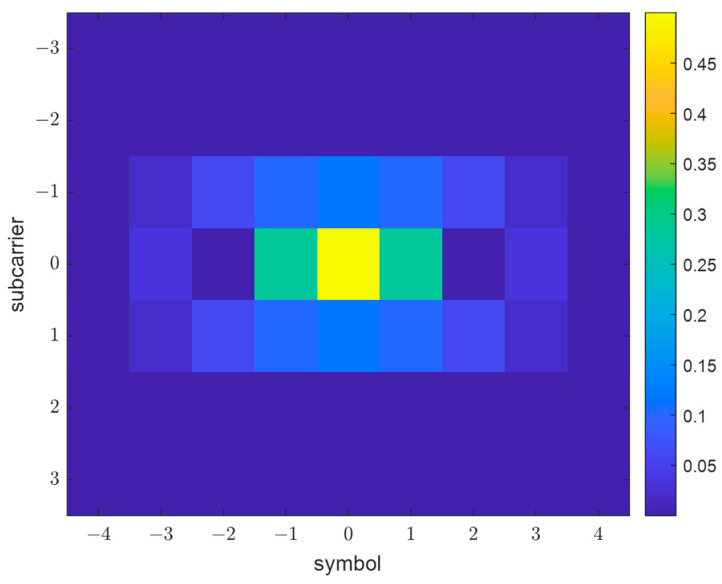
PHYDYAS prototype filter ambiguity function.

Obviously, the strict observance of these conditions for physically realizable filters is impossible; therefore, the scientific community has performed work on the synthesis of quasi-optimal prototype filter designs that are close to the conditions of perfect reconstruction. These prototypes differ mainly in the properties of the frequency (determining the level of distortion) and time localization. As part of a comparative analysis carried out by the PHYDYAS research project, the filter presented in the work by Bellanger [18] and later called the PHYDYAS prototype filter was adopted for use. This filter provides good localization in the frequency domain and low distortion at a relatively short IR length (numerically equal to the number of channels times 4), which can be seen in the discrete ambiguity function of this prototype filter $A_{g}$ presented in Figure 1, defined as:

(2) $$\begin{array}{c} A_{g}\left(\tau ,\mu \right)=\int_{-\infty }^{\infty }g\left(t+\frac{\tau }{2}\right)g^{*}\left(t+\frac{\tau }{2}\right)e^{-j2\pi \mu t}dt\\ ⇓\\ A_{g}\left[n,m\right]=\sum_{i}g\left[i+\frac{n}{2}\right]g^{*}\left[i-\frac{n}{2}\right]e^{-\frac{j2\pi im}{M}} \end{array}$$

FBMC-OQAM uses a computationally efficient algorithm analysis and synthesis filter banks based on the modified discrete Fourier transform (MDFT) filter bank presented in [19]. This algorithm allows the use of subchannels twice as wide in frequency, but the perfect reconstruction conditions are met only in the real part of the signal, and in the imaginary part, there is a pseudo-noise composition equal to the sum of the surrounding symbols, with weights determined by the ambiguity function of the prototype filter [20]. The application of this algorithm for information transmission was presented in [4], where it was shown that the shift of the real and imaginary parts of the QAM symbol modulated using the MDFT synthesis bank (in-phase and quadrature components of the symbol are transmitted using two pulse-amplitude-modulated (PAM) symbols with a twofold shorter duration and shift relative to each other), as was shown in [21], allows for a lower level of self-interference. Thus, the analytical expression for the FBMC-OQAM group signal can be written as:

(3) $$s\left(k\right)=\sum_{m=0}^{M-1}\sum_{n\in Z}a_{m,n}g\left(k-nN\right)e^{j\frac{2\pi }{M}m\left(k-\frac{L_{g}-1}{2}\right)}e^{j\phi_{m,n}}$$

where $a_{m,n}$ denotes transmitted PAM symbols, which are obtained by extracting the real and imaginary parts from QAM symbols, N=M/2 is a discrete shift in the time domain by half the length of the baseband symbol, $L_{g}$ is the filter prototype IR length, $\phi_{m,n}=\frac{\pi }{M}\left(m+n\right)$ is the phase shift for the m-th subcarrier and n-th symbol, and M is the number of subcarriers.

**Figure 2:**
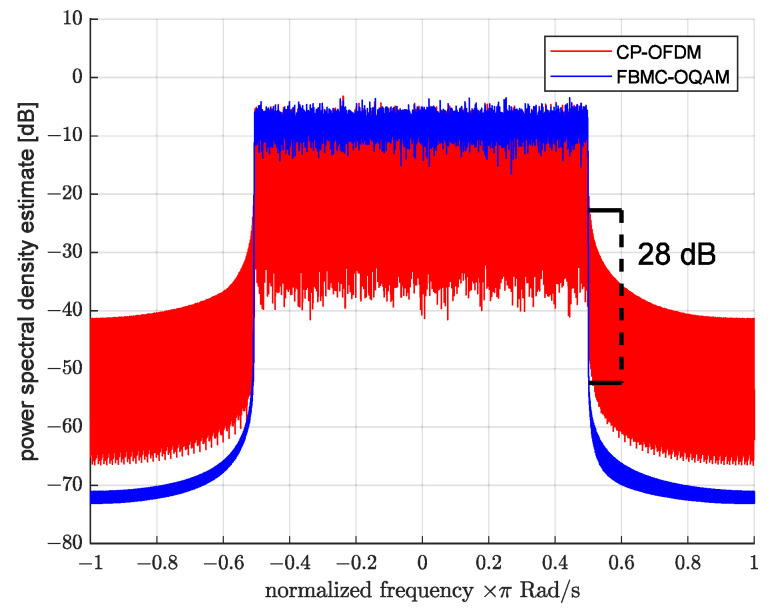
FBMC-OQAM and CP-OFDM power spectral density comparison.

Thanks to the filtration of subcarrier signals, the FBMC-OQAM group signal has significantly better power spectral density (PSD) localization compared to CP-OFDM. To clearly demonstrate this difference, two signals were generated using each of the technologies, where half of the subcarriers located in the center of the main frequency range contained random data symbols (the same for both signals), and the symbols of the remaining subcarriers were equal to zero. The total number of subcarriers was set to 512. The power spectral densities of these signals are presented in Figure 2, which shows that for two subcarriers offset from useful signal frequencies, FBMC-OQAM provides a 28 dB lower out-of-band signal level compared to CP-OFDM.

The demodulated symbols are in turn calculated as:

(4) $${\hat{a}}_{m,n}=\sum_{k=0}^{\infty }\sum_{m=0}^{M-1}y\left[k-\frac{nM}{2}\right]g\left[\frac{nM}{2}-k\right]e^{-\frac{j2\pi m\left(k-\frac{L_{g}-1}{2}\right)}{M}}e^{-j\phi_{m,n}},$$

where y is the received baseband signal.

**Figure 3:**
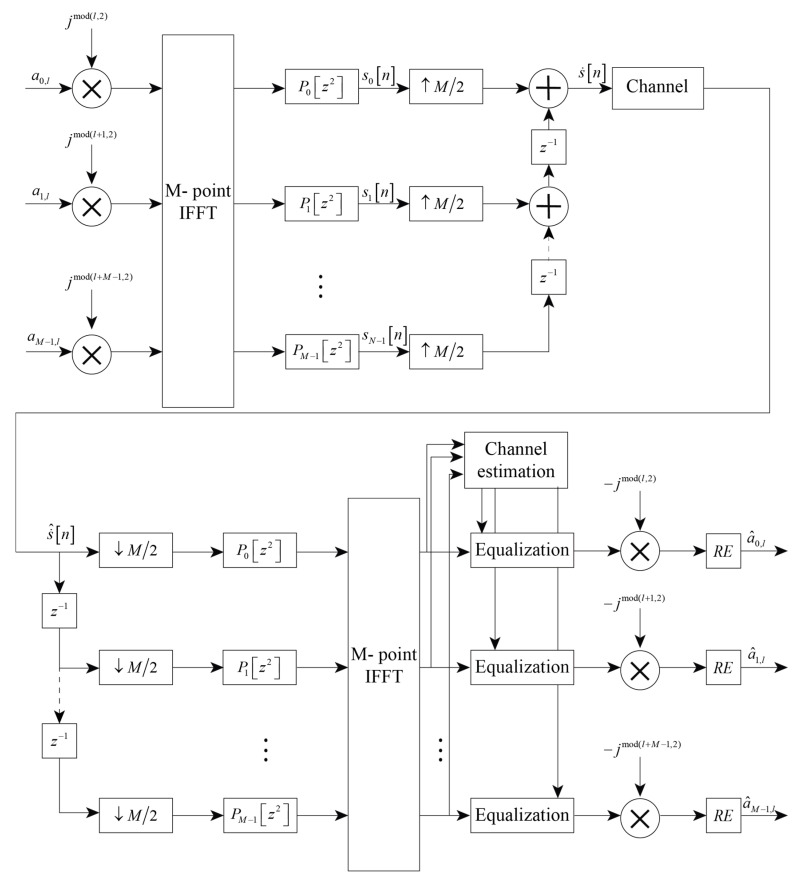
FBMC-OQAM data transmission model.

Thus, the block scheme of the described model, including the equalization and selection of the symbol’s real part, can be represented as shown in Figure 3. Data symbols are multiplied by the phase shift factor $\phi_{m,n}$, and then subcarrier signals become frequency-modulated in the inverse Fourier transform. Further narrowband channels are filtered by the set of synthesis filters with transfer functions $G_{0}\left[z\right],G_{1}\left[z\right],\ldots ,G_{M-1}\left[z\right]$, corresponding to impulse responses $g\left(k-nN\right),g\left(k-nN\right)e^{j\frac{2\pi }{M}\left(k-\frac{L_{g}-1}{2}\right)},\ldots ,g\left(k-nN\right)e^{j\frac{2\pi (M-1)}{M}\left(k-\frac{L_{g}-1}{2}\right)}$, whose output signals are multiplexed in the modulated time-domain FBMC signal. The demodulation part works similarly but in reverse order: demultiplexing of the time-domain signal, filtering with a set of analysis filters (which, as was shown in [9], are equivalent to synthesis filters for the FMBC system), frequency demultiplexing by the FFT transform, the cancellation of the additional phase shift, the equalization stage and, finally, the separation of the received symbol’s real part, which holds transmitted information.

At the same time, despite the fact that information is contained only in the real part of the symbols, in the case of a change in the phase of the symbols when passing through the channel, the imaginary part must be taken into account when estimating the channel and synchronization [22], since in the presence of phase shifts, the imaginary part of the interference affects the real part.

## 3. MIMO Tapped Delay Line Channel Model

A MIMO channel can be described using a channel matrix, each element of which corresponds to one of the propagation paths between the transmitting and receiving antennas, in which case the MIMO channel with memory can be described by a three-dimensional matrix:

(5) $$\begin{array}{cc} H\left(l\right)=\left[\begin{array}{ccc} h_{11}\left(l\right) & \ldots & h_{N_{T}1}\left(l\right)\\ \vdots & \ddots & \vdots \\ h_{1N_{R}}\left(l\right) & \ldots & h_{N_{R}N_{T}}\left(l\right) \end{array}\right] & l=0,1,\ldots ,L \end{array},$$

where $N_{T}$ and $N_{R}$ are the number of transmitting and receiving antennas, respectively, and L is the channel impulse response length in the samples.

In this case, the propagation of the signal through the channel is expressed mathematically as the multiplication of the transmitted signal vector by the channel matrix for each beam. For tapped delay line channels, the expression for the received FBMC-OQAM MIMO signal, provided that it passes through the channel with memory, is defined as:

(6) $$Y\left(k\right)=\sum_{l=0}^{L-1}H\left(l\right)S\left(k-l\right)+n\left(k\right),$$

where $Y\left(k\right)={\left[y_{1}\left(k\right),y_{2}\left(k\right),\ldots ,y_{N_{R}}\left(k\right)\right]}^{T}$ is the column vector of received signals, $S\left(k\right)=\left[s_{1}\left(k\right),s_{2}\left(k\right),\ldots ,s_{N_{T}}\left(k\right)\right]$ is the row vector of transmitted signals, $n\left(k\right)={\left[n_{1}\left(k\right),n_{2}\left(k\right),\ldots ,n_{R}\left(k\right)\right]}^{T}$ is the noise vector, where $n_{i}\left(k\right)$ is the zero-mean white Gaussian noise, and k is the discrete-time index.

## 4. Real and Imaginary Parts of Interference Correlation

For further analysis, we present the expression for the symbols at the demodulator input, taking into account the MIMO channel, by substituting Equation (3) into Equation (6):

(7) $${\hat{A}}_{m_{0},n_{0}}=\sum_{l=0}^{L-1}\sum_{m=0}^{M-1}\sum_{n\in Z}A_{m,n}H\left(l\right)g\left(k-l-nN\right)e^{j\frac{2\pi }{M}m\left(k-l-\frac{L_{g}-1}{2}\right)}e^{j\phi_{m,n}}+n\left(k\right),$$

where $A_{m_{0},n_{0}}=\left[a_{1,m_{0},n_{0}},a_{2,m_{0},n_{0}},\ldots ,a_{Tr,m_{0},n_{0}}\right]$ is a row vector containing the characters to be transmitted by PAM.

By separating the useful and interference parts of Equation (7) based on Equation (3), the symbols at the demodulator output before taking the real part and without considering noise n(k) can be represented as:

(8) $${\hat{A}}_{m_{0},n_{0}}=A_{m_{0},n_{0}}{\tilde{H}}_{m_{0}}^{0,0}+I_{m_{0},n_{0}},$$

where the component $A_{m_{0},n_{0}}{\tilde{\tilde{H}}}^{0,0}$ describes the useful part of the symbol evaluation, taking into account the influence of the channel and the signal passing through the prototype filter with a delay, and $I_{m_{0},n_{0}}$ specifies the interference component, determined by the influence of all surrounding symbols on the given one:

(9) $$I_{m_{0},n_{0}}=\sum_{p,q\neq \left(0,0\right)}{\left(-1\right)}^{n_{0}p}A_{m_{0}+p,n_{0}+q}{\tilde{H}}_{m_{0}}^{p,q},$$

where the matrix ${\tilde{H}}_{m_{0}}^{p,q}$ is the FBMC-OQAM system transmultiplexer response describing the effect of a symbol with a shift on p symbols and q subcarriers from the $m_{0},n_{0}$ symbol appearing due to a non-ideal separation by a prototype filter for rays arriving with a delay:

(10) $${\tilde{H}}_{m_{0}}^{p,q}=\left(\begin{array}{ccc} \sum_{l=0}^{L-1}h_{1,1}\left(l\right)A_{g}\left(-qN-l,p\right)e^{-j\frac{2\pi }{M}m_{0}l} & \ldots & \sum_{l=0}^{L-1}h_{N_{T},N_{R}}\left(l\right)A_{g}\left(-qN-l,p\right)e^{-j\frac{2\pi }{M}m_{0}l}\\ \vdots & \ddots & \vdots \\ \sum_{l=0}^{L-1}h_{N_{T},1}\left(l\right)A_{g}\left(-qN-l,p\right)e^{-j\frac{2\pi }{M}m_{0}l} & \ldots & \sum_{l=0}^{L-1}h_{N_{T},N_{R}}\left(l\right)A_{g}\left(-qN-l,p\right)e^{-j\frac{2\pi }{M}m_{0}l} \end{array}\right)$$

After removing the channel effect using an ideal single-tap equalizer, calculated by the zero-forcing (ZF) criterion as:

(11) $$B_{m_{0}}={\left({\left[{\tilde{H}}_{m_{0}}^{0,0}\right]}^{T}{\tilde{H}}_{m_{0}}^{0,0}\right)}^{-1}{\left[{\tilde{H}}_{m_{0}}^{0,0}\right]}^{T},$$

the demodulated signal can be expressed as:

(12) $${\hat{Z}}_{m_{0},n_{0}}=A_{m_{0},n_{0}}+J_{m_{0},n_{0}}$$

where $J_{m_{0},n_{0}}$, similarly to Equation (9), is equal to

(13) $$J_{m_{0},n_{0}}=\sum_{\begin{array}{l} pq\\ \neq \left(0,0\right) \end{array}}{\left(-1\right)}^{n_{0}p}A_{m_{0}+p,n_{0}+q}{\tilde{H}}_{m_{0}}^{p,q}B_{m_{0}}$$

Based on Equation (13), it is possible, with known true symbol values A, to calculate and compensate for the intrinsic interference that occurs in the FBMC-OQAM modem.

The pseudoinverse matrix calculation in Equation (11) requires ${\tilde{H}}_{m_{0}}^{0,0}$ to be a full row-rank matrix. For further analysis, we assume that ${\tilde{H}}_{m_{0}}^{0,0}$ meets these conditions.

However, in practice, the true values of A are unknown, which requires a different way to estimate the intrinsic interference. Obviously, when using the real PAM modulation, after eliminating the influence of the channel, the imaginary part of the symbol estimate ${\hat{z}}_{m_{0},n_{0}}$ will consist only of the imaginary part of the intrinsic interference $J_{m_{0},n_{0}}$; therefore, it can be easily defined in the receiver as $ℑ\left[{\hat{z}}_{m_{0},n_{0}}\right]$. In [7], it was first established that for the single-input–single-output (SISO) channel, there is a correlation between the real part of $J_{m_{0},n_{0}}$ and the imaginary part of $J_{m_{0},n_{0}\pm t}$, where t is a nonzero time shift. Thus, knowing the imaginary part of the interference and the correlation between the real and imaginary parts, it becomes possible to estimate the real part. To do this, we define the unnormalized correlation matrix between the real part of $J_{m_{0},n_{0}}$(Jm0,n0Re) and the imaginary part of $J_{m_{0},n_{0}}$ (Jm0,n0Im) for the given channel H(l) as:

(14) C(t,m0)=E[(Jm0,n0Re)TJm0,n0−tIm]=σa2INT×NT∑p,q≠(0,0)≠(0,t)(−1)pt[Re(H˜m0p,qBm0)]TIm(H˜m0p,q−tBm0)

Since the mathematical expectation of the real and imaginary components of internal noise is zero, M[Jm0,n0Re]=M[Jm0,n0Im]=0 (based on Equation (13) and equality to zero M[A]), and their variances are, respectively, equal to D[Jm0,n0Re]=σRPI2IR×R and D[Jm0,n0Im]=σIPI2IR×R, where $I_{R\times R}$ is an identity matrix of size R×R.

The variances $\sigma_{IPI}^{2}$ and $\sigma_{RPI}^{2}$, considering that M[Jm0,n0Im]=M[Jm0,n0Re]=0, equal

(15) σRPI2ITr×Tr=M[(Jm0,n0Re)2]=σa2ITr×Tr∑p,q≠(0,0)≠(0,t)[Re(H˜m0p,qBm0)]TRe(H˜m0p,qBm0)

(16) σIPI2ITr×Tr=M[(Jm0,n0Im)2]=σa2ITr×Tr∑p,q≠(0,0)≠(0,t)[Im(H˜m0p,qBm0)]TIm(H˜m0p,qBm0)

Thus, the normalized correlation coefficient can be defined as:

(17) $$\rho \left(t,m_{0}\right)=\frac{C\left(t,m_{0}\right)}{\sigma_{RPI}\sigma_{IPI}}$$

**Figure 4:**
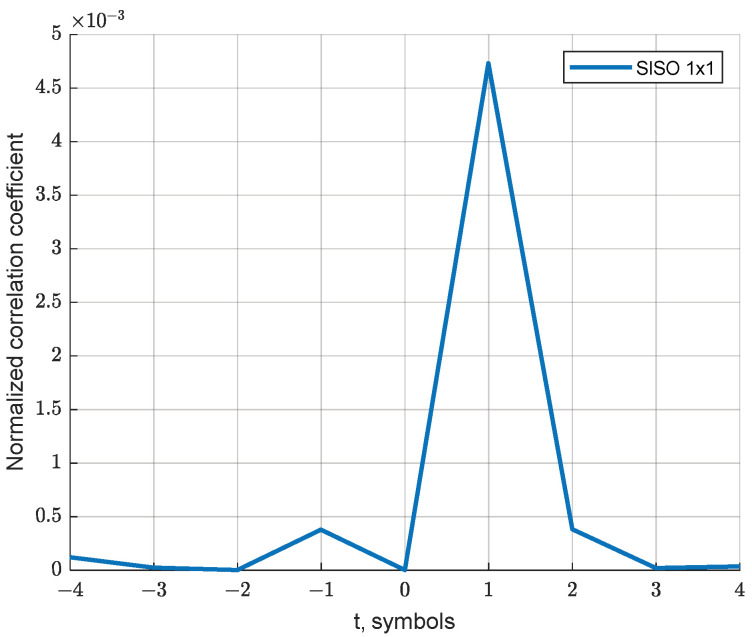
Normalized self-interference correlation coefficient versus time shift.

Figure 4 shows the dependence of the normalized correlation coefficient ρ on t for the 2 × 2 MIMO scheme, showing the presence of a theoretical relationship between the nearest FBMC-OQAM symbols.

## 5. Real Part of the Interference Cancellation

Knowing the correlation relationship between the real and imaginary parts of the interference, as well as the fact that the imaginary part of the received symbols contains only the interference component, we can estimate the value of the real part of the interference Jm0,n0Re as:

(18) J^m0,n0Re=∑tρ(t,m0)ℑ[Z^m0,n0+t]

In [16], the authors showed that the correlation between adjacent subcarriers appears only in the presence of a Doppler shift in the frequency of the received signal. Since this work is focused on the application in ionospheric shortwave communication systems, where the influence of the Doppler shift is minimal [11], and the gain shown by the authors manifested itself at Doppler factor values greater than 0.1, we will restrict ourselves to considering only a multipath channel with memory. Based on the values of the determinants of the correlation matrices for various shifts between the symbols used for the interference estimation, as presented in Figure 3, the set of time shifts for symbols used in Equation (16) can be limited by the values t=−1 and t=1. Then, the expression for estimating the real part of the interference takes the form:

(19) J^m0,n0Re=ρ(1,m0)ℑ[Z^m0,n0+1]+ρ(−1,m0)ℑ[Z^m0,n0−1]

The FBMC-OQAM MIMO system signal demodulation algorithm, taking into account the proposed algorithm, can be rewritten as:

(20) a^m0,n0=Re{ym0,n0H˜˜m0−1}−J^m0,n0Re

**Figure 5:**
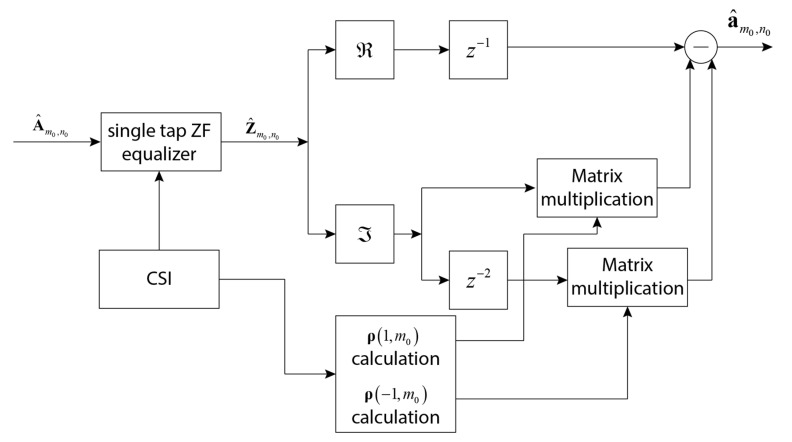
FBMC-OQAM MIMO ERIP equalizer block scheme.

The block diagram of the FBMC-OQAM MIMO equalizer after calculating $y_{m_{0},n_{0}}$ with internal noise compensation is shown in Figure 5.

## 6. Computational Complexity

The computational cost required for the operation of the ERIP algorithm consists of two components: the first includes the calculation of the normalized correlation coefficients of the real and imaginary parts of the residual interference of PAM symbols, and the second includes online operations to compensate for the real part of the interference. The complexity of the online computations of the ERIP algorithm is $M\left(N_{T}^{2}n_{t}+N_{R}N_{T}\right)$ real multiplications, where $n_{t}$ is the number of time shifts for which the correlation coefficient is calculated, which, for the 2 × 2 MIMO scheme, gives 12 real additions and multiplications per symbol at $n_{t}$ = 2. The complexity of calculating the correlation coefficients of the real and imaginary parts of the interference when using the pruned FFT algorithm and the symmetry property of the prototype filter uncertainty function to calculate the value of Equation (17) can be defined as:

(21) $$RM_{ERIP}=N_{T}N_{R}\left(M\log_{2}L_{h}-4L_{h}\right)\frac{\#\left(p,q\right)-1}{4}\#\left(p,q\right),$$

where #(p,q) denotes the number of elements of the set (p,q), which, based on the prototype filter ambiguity function [18] shown in Figure 1, can be limited to (|p|<1,|q|<3), and the cardinality of the set (p,q) will be equal to 21.

**Figure 6:**
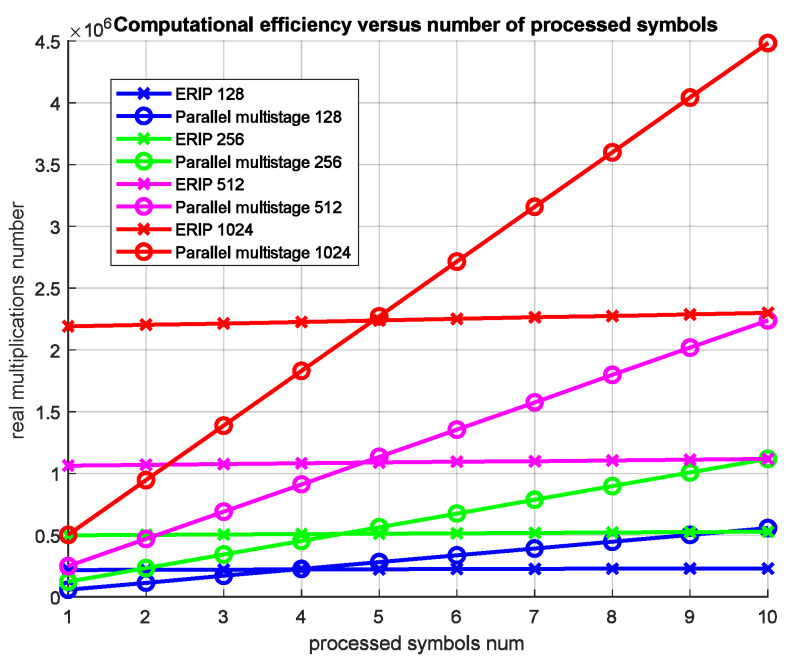
ERIP and parallel multistage computational efficiency versus number of processed symbols and subcarriers.

For comparison, the computational complexity of the parallel multistage equalizer, the implementation of which for the MIMO channel was presented in [23], is:

(22) $$RM_{PM}=MK_{R}\left(N_{R}\left(\log_{2}M-1\right)+\left(\kappa +2\right)N_{R}+3N_{S}N_{R}(L_{h}-1)\right),$$

where κ is the filter prototype overlapping factor, and $K_{R}$ is the number of parallel stages. Thus, for the number of subcarriers equal to 128 to 1024, $N_{T}=N_{R}=2$, $L_{h}=37$, κ=4 and $K_{R}=2$, the number of operations performed depends on the number of processed symbols for the presented equalization algorithms; it can be observed that with an increase in the number of processed ERIP symbols, the equalizer shows itself to be more preferable (Figure 6) starting from 4–5 symbols, depending on the number of subcarriers. At the same time, when using the calculated correlation coefficients for more than 10 FBMC-OQAM symbols, the ERIP gain becomes approximately twofold. In this case, such a number of processed symbols corresponds to the required channel coherence time, ranging from 3.2 ms to 25.6 ms, with the number of subcarriers ranging from 128 to 1024 and a signal bandwidth of 400 kHz, which, for example, for an HF channel, does not exceed the coherence time [24].

## 7. Results of Computer Modeling

To check the efficiency of the presented MIMO ERIP equalizer, a comparative simulation was carried out under the conditions of a stationary ionospheric shortwave multipath channel implemented in accordance with the recommendation ITU-R F.1487 [18] for the case of low-latitude quiet conditions and high-latitude quiet conditions for a system with two transmitting and two receiving antennas. The signal bandwidth was taken to be 500 kHz, the number of subcarriers was 128, and the spacing between subcarriers was 320 Hz.

**Figure 7:**
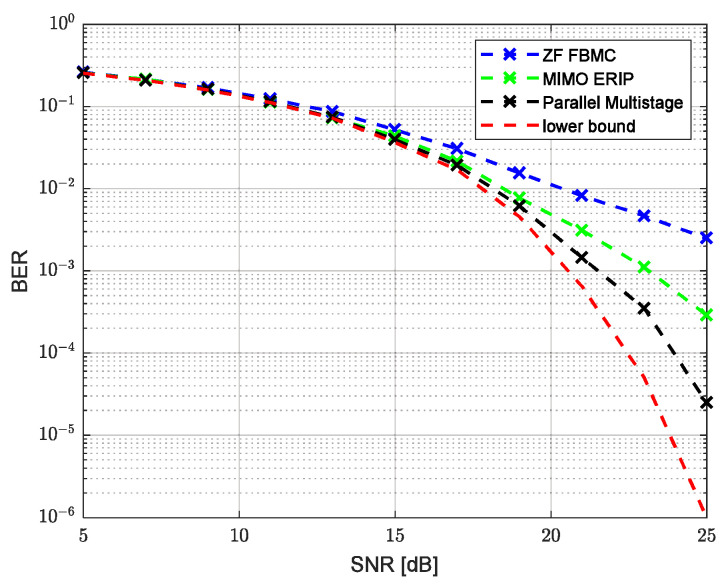
Uncoded BER performance versus SNR for HFHQ channel model.

**Figure 8:**
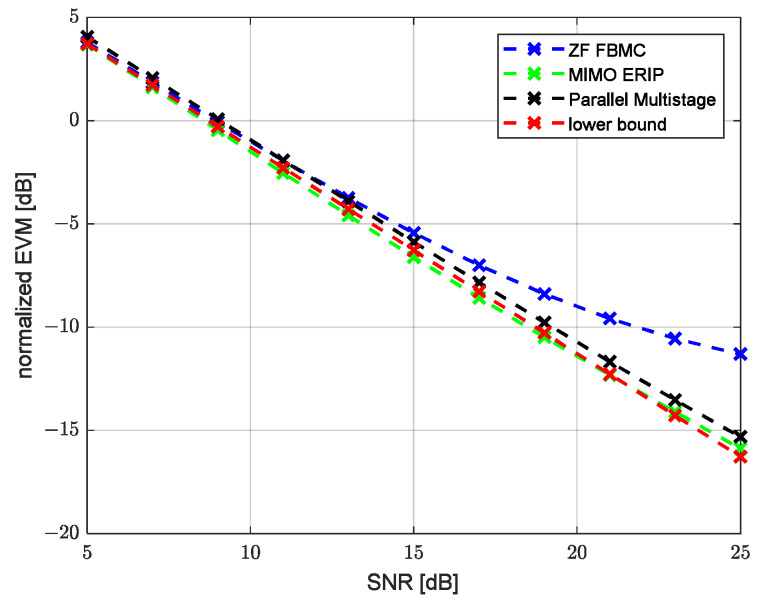
EVM performance versus SNR for HFHQ channel model.

Modeling was performed for two channels’ parameter combinations, corresponding to an HF low-latitude channel with quiet conditions (HFLQ) and a high-latitude channel with quiet conditions (HFHQ). The first channel model corresponds to two beams with equal average power and a delay between beams of 1 ms. Figure 7 and Figure 8 show the dependence of the uncoded bit error ratio (BER) and the error vector amplitude (EVM) over the SNR, respectively, for the classic single-tap FBMC-OQAM ZF equalizer, MIMO ERIP equalizer, parallel multistage equalizer for the HFHQ channel model and the theoretical minimum for the case without the channel influence. Based on the presented dependencies, we can observe the close performance values of the MIMO ERIP algorithm compared to the parallel multistage equalizer, which, taking into account the lower computational complexity, makes the ERIP-based algorithm the preferred option for systems that are critical to the computational complexity of the implementation of the receiving path. The performance loss at high SNR values can be explained by the imperfect calculation of the real and imaginary parts of the interference correlation coefficient due to prototype filter ambiguity function truncation by one surrounding subcarrier and three surrounding symbols.

**Figure 9:**
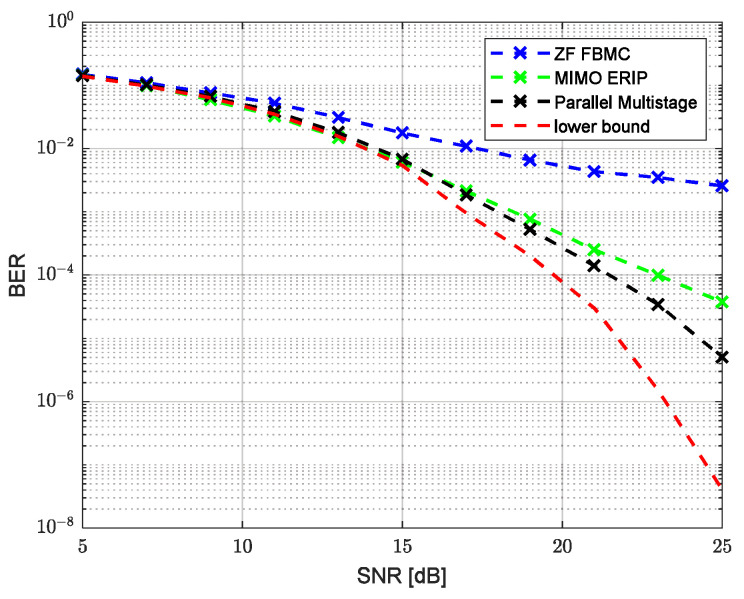
Uncoded BER performance versus SNR for HFLQ channel model.

**Figure 10:**
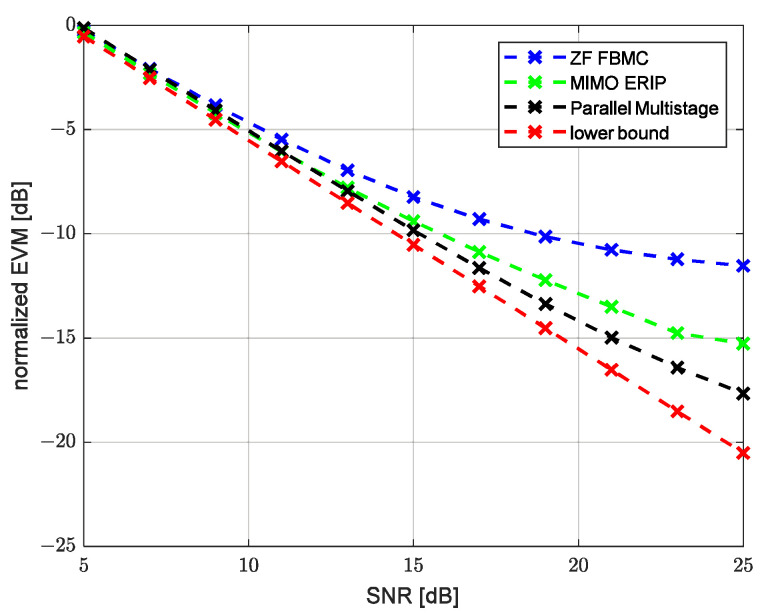
EVM performance versus SNR for HFLQ channel model.

Figure 9 and Figure 10 show the dependence of the uncoded BER and EVM over the SNR for the same algorithms as in the previous case but in HFLQ channel conditions. For these conditions, the channel impulse response can be represented by two beams with equal power and a delay between them of 0.5 ms. The HFLQ channel has less difficult conditions, which explains the better performance of all presented algorithms, but we can see that the difference between MIMO ERIP and parallel multistage algorithms grows, which can also be explained by the growing impact of residual self-interference.

## 8. Conclusions

In this work, we have expanded the idea of equalization with real interference prediction for MIMO channels. For this, a novel mathematical analysis of FBMC-OQAM self-interference parts’ correlation in MIMO channels with memory was presented. Based on this analysis, a method was presented for calculating the normalized correlation coefficient between the real and imaginary parts of the intrinsic interference of adjacent symbols caused by the processing of the rays of the received signal by a set of analysis filters based on a prototype with a non-ideal ambiguity function. Additionally, for the calculated correlation coefficients, the highest correlation was shown for the nearest symbols, which is in accordance with the results obtained earlier for this method for frequency and doubly dispersive SISO channels. A comparison of the computational complexity of the proposed algorithm and the parallel multistage equalizer was shown, as a result of which it was shown that for channels that allow using the same correlation coefficients for five or more FBMC-OQAM symbols, the proposed scheme is more computationally efficient. However, the complexity of the correlation coefficient calculation makes the usage of the proposed algorithm impractical in fast-fading channels. At the same time, the results of the simulation modeling of the performance of these algorithms according to the BER and EVM criteria for the ionospheric HF channel showed only a slight loss for the MIMO ERIP algorithm compared to the parallel multistage equalizer, which suggests its applicability for MIMO systems, which require better-quality equalization of the received signals but are critical to the computational complexity of the equalizer.

## Data Availability

Not applicable.
